# Does Intensive Treatment Select for Praziquantel Resistance in High-Transmission Settings? Parasitological Trends and Treatment Efficacy Within a Cluster-Randomized Trial

**DOI:** 10.1093/ofid/ofaa091

**Published:** 2020-03-12

**Authors:** John Vianney Tushabe, Lawrence Lubyayi, Joel Sserubanja, Prossy Kabuubi, Elson Abayo, Samuel Kiwanuka, Jacent Nassuuna, James Kaweesa, Paul Corstjens, Govert van Dam, Richard E Sanya, William Ssenyonga, Edridah Muheki Tukahebwa, Narcis B Kabatereine, Alison M Elliott, Emily L Webb, LaVIISWA trial team, Richard Sanya, Margaret Nampijja, Harriet Mpairwe, Barbara Nerima, Joel Serubanja, Emily Webb, Lawrence Lubyayi, Hellen Akurut, Justin Okello, Sebastian Owilla, Jacob Ochola, Christopher Zziwa, Milly Namutebi, Esther Nakazibwe, Josephine Tumusiime, Caroline Ninsiima, Susan Amongi, Grace Kamukama, Susan Iwala, Rita Asherwin, Rehema Nampijja, Florence Akello, Mirriam Akello, Robert Kizindo, Moses Sewankambo, Denis Nsubuga, Stephen Cose, Prossy Kabuubi Nakawungu, Emmanuel Niwagaba, Gloria Oduru, Grace Kabami, Elson Abayo, Fred Muwonge Kakooza, Joyce Kabagenyi, Gyaviira Nkurunungi, Angela Nalwoga, John Vianney Tushabe, Jacent Nassuuna, Bridgious Walusimbi, David Abiriga, Richard Walusimbi, Cynthia Kabonesa, James Kaweesa, Edridah Tukahebwa, Moses Kizza, Alison Elliott

**Affiliations:** 1 Immunomodulation and Vaccines Research Programme, Medical Research Council/Uganda Virus Research Institute and London School of Hygiene and Tropical Medicine Uganda Research Unit, Entebbe, Uganda; 2 Wellcome Sanger Institute, Wellcome Trust Genome Campus, Hinxton, United Kingdom; 3 Department of Epidemiology and Biostatistics, School of Public Health, University of the Witwatersrand, Johannesburg, South Africa; 4 Vector Control Division, Ministry of Health, Kampala, Uganda; 5 Leiden University Medical Center, Leiden, the Netherlands; 6 Department of Internal Medicine, College of Health Sciences, Makerere University, Kampala, Uganda; 7 Department of Clinical Research, London School of Hygiene and Tropical Medicine, London, UK; 8 MRC Tropical Epidemiology Group, London School of Hygiene and Tropical Medicine, London, UK

**Keywords:** drug resistance, mass drug administration, praziquantel, schistosomiasis

## Abstract

**Background:**

Praziquantel mass drug administration (MDA) is recommended in schistosomiasis-endemic areas. Animal models demonstrate *Schistosoma* parasite resistance to praziquantel after repeated exposure.

**Methods:**

We conducted a parasitological survey in 26 fishing communities in Uganda after 4 years of quarterly (13 communities) or annual (13 communities) praziquantel MDA, with *Schistosoma* infection detected by single-stool-sample Kato-Katz. A test of cure was done in participants who were positive on both urine circulating cathodic antigen test and 3-sample Kato-Katz. We calculated cure rates (CRs) and egg reduction rates (ERRs) based on 3-sample Kato-Katz and infection intensity using worm-specific circulating anodic antigen (CAA) in blood, comparing these between quarterly and annually treated participants.

**Results:**

Single-sample Kato-Katz *Schistosoma mansoni* prevalence was 22% in 1,056 quarterly treated participants and 34% in 1,030 annually treated participants (risk ratio, 0.62; 95% confidence interval [CI], 0.40 to 0.94). Among 110 test-of-cure participants, CRs were 65% and 51% in annually and quarterly treated villages, respectively (odds ratio, 0.65; 95% CI, 0.27 to 1.58); ERRs were 94% and 81% (difference, –13%; 95% CI, –48% to 2%). There was no impact of quarterly vs annual praziquantel on *S. mansoni* by CAA.

**Conclusions:**

In this schistosomiasis hot spot, there was little evidence of decreased praziquantel efficacy. However, in the absence of alternative therapies, there remains a need for continued vigilance of praziquantel efficacy in the MDA era.

Schistosomiasis affects >250 million people worldwide, mostly in sub-Saharan Africa [1, 2]. Praziquantel is the only drug that is widely available for the treatment of *Schistosoma mansoni* [3]. It is used as single-dose mono-therapy in mass drug administration (MDA) programs for the control of schistosomiasis in all endemic settings [4]. However, ~10%–20% of infected people are not cured after single-dose treatment [5], depending on initial infection intensity.

In Uganda, as in other schistosomiasis-endemic African countries [6], MDA aims to reduce the intensity of infection and hence pathology. Permanent cure and, ultimately, elimination are not expected in the absence of additional interventions [7, 8]. Individuals receiving MDA in high-transmission communities are repeatedly exposed to re-infection due to ongoing contamination of water and presence of the snail vector, in conjunction with contributing factors such as poor sanitation. MDA programs alone have been shown to be insufficient for effective control of schistosomiasis [9]; other interventions such as WASH (sanitation and personal hygiene) and disruption of the parasite life cycle/snail control remain key in combatting this health problem. Delivery of such interventions in affected communities is hampered by factors such as the unsuitability of introducing molluscicides in large water bodies such as Lake Victoria, lack of specific funds to establish good sanitation facilities such as toilets and safe domestic water systems, and challenges in building such facilities in informal settlements on sandy lake shores. Hence, MDA continues to be the primary weapon in such communities in the fight against this global burden.

The continued use of praziquantel as monotherapy in high-transmission communities may lead to the development of drug resistance [10], a major international concern. Variation in susceptibility of *Schistosoma* species to praziquantel treatment has been observed between different human populations [11], and studies in mice have demonstrated the potential for inducing resistance through drug pressure [12–14].

Among participants of the Lake Victoria Island Intervention Study on Worms and Allergy-related diseases (LaVIISWA) [15], we conducted a parasitological survey after 4 years of anthelminthic MDA intervention. For a subgroup of individuals who were infected in this survey, we administered praziquantel treatment and assessed cure rate 4 weeks later, comparing those who had received quarterly praziquantel treatment with those who had received annual praziquantel treatment over the preceding 4 years.

## METHODS

### Study Setting

The study was conducted in fishing communities located in the Koome islands of Lake Victoria, Uganda, a remote setting accessible within 2–3 hours from the mainland by powered canoe. Schistosomiasis is endemic in the study area, with prevalence based on Kato-Katz analysis of a single stool sample estimated at 52% in the LaVIISWA baseline survey in 2012–2013 (72% prevalence by the more sensitive urine circulating cathodic antigen [CCA] assay) [16]. Soil-transmitted helminths are also present but with lower prevalence, with 2012–2013 estimates of 22% for hookworm (based on the more sensitive polymerase chain reaction test), 10% for *Trichuris*, and 1% for *Ascaris* (both based on Kato-Katz). Sanitation facilities are limited, with few households having access to toilets or to water sources other than the lake. Community members generally derive their livelihood from the lake, and most of the activities on these islands are related to fishing. Almost all water for domestic use is obtained from the lake, with 1 of the villages having a spring and another having piped water. Hence there is constant exposure of village occupants to cercaria, leading to frequent re-infections following effective treatment. The communities are comprised of temporary wooden structures on land belonging to “landlords,” and the population is highly mobile, both between and within island villages and between islands and the mainland.

### Study Design

The LaVIISWA trial (ISRCTN47196031) was originally designed to assess the effect of 3 years’ intensive vs standard anthelminthic treatment on allergy-related outcomes and helminth-related pathology. Twenty-six fishing communities were randomized in a 1:1 ratio to receive either standard or intensive anthelminthic intervention. Standard intervention comprised annual praziquantel plus twice-yearly single-dose albendazole, whereas intensive intervention comprised quarterly praziquantel with use of the extended 60-cm height pole [17] to allow treatment of preschool-aged children, for whom tablets were crushed and given in juice, plus quarterly triple-dose albendazole. A pre-intervention baseline survey and 3-year outcome survey were conducted, with smaller interim parasitological surveys conducted after 1 and 2 years of the intervention. Full details of the original trial design [16] and results of the outcome survey conducted after 3 years of intervention [15] have been reported.

To assess longer-term effects of MDA on infection prevalence and to investigate the possibility of induction of, or selection for, praziquantel resistance, the LaVIISWA interventions and follow-up were extended for another year, and at the end of 4 years of intensive vs standard treatment, a parasitological survey was conducted across all study villages, and a formal test of cure was done among selected participants with persistent infection from each trial arm.

### Study Participants

For the parasitological survey, 70 households were randomly selected from each of the 26 fishing communities. All residents of selected households were invited to participate in the survey, and each was asked to provide a stool sample. After samples had been collected, participants taking part in the parasitological survey were treated under observation with a single dose of praziquantel at 40 mg/kg (estimated by height pole), in accordance with the trial MDA allocation.

For the test-of-cure substudy, which was done in parallel with the parasitological survey, 8 communities were selected pragmatically, with regard to logistics; 4 had received standard intervention, and 4 had received intensive intervention. Within these villages, treatment registers were used to identify individuals who had been resident in the same village throughout the 4-year intervention period and who had received at least 50% of the expected praziquantel treatments during the trial. Households containing these individuals were approached in a random order, and all eligible individuals in these households were invited to take part, until the target sample size was achieved. Individuals who were not selected for the test-of-cure substudy continued to undergo the standard procedures for the parasitological survey. From those selected, information about age, gender, history of residence in the village, and exposure to lake water was obtained, and a screening urine sample was requested to test for *Schistosoma* infection using a point-of-care CCA test (Rapid Medical Diagnostics). Participants who tested positive on CCA were requested to provide stool samples on 3 consecutive days for analysis by the Kato-Katz method, and a blood sample was taken from each of these participants for circulating anodic antigen (CAA). After all the samples had been collected, participants were treated under observation with a single dose of praziquantel at 40 mg/kg. Participants found to be Kato-Katz positive at enrollment into the test-of-cure substudy based on these 3 stool samples were followed up for 4 weeks and requested to provide a further 3 stool samples on consecutive days and a further blood sample for serum CAA in order to assess cure rates, egg reduction rates, and infection intensity based on CAA. At follow-up, after all sample collection was done, if an individual still had the infection, they were treated again with praziquantel.

### Diagnostic and Laboratory Methods

For the parasitological survey and for repeated samples at baseline and after 4 weeks in the test-of-cure substudy, the stool Kato-Katz technique was used to assess for *Schistosoma mansoni* infection. Two slides were prepared immediately from stool samples received following the procedures as previously described [18], and each slide was read by a different experienced technician to ensure accuracy.

The urine CCA test was used for qualitative detection of *Schistosoma mansoni* infection at enrollment into the test-of-cure substudy. Each participant was asked to provide a midstream urine sample in a sterile plastic container for the CCA test. The CCA was performed according to the kit manufacturer’s instructions (Rapid Medical Diagnostics).

We further used the sensitive *Schistosoma* up-converting phosphor lateral flow circulating anodic antigen (UCP-LF-CAA) to quantify the CAA in sera collected at baseline and 4 weeks post-treatment from participants in the test-of-cure substudy. The applied test format (SCAA20) allowed detection of CAA down to a level of 10 pg/mL. Twenty-five samples that showed inconsistent results between the Kato-Katz technique and SCAA20 were repeated with a higher-sensitivity test format (SCAA500), allowing detection down to 1 pg/mL [19, 20].

### Ethical Considerations

Ethical approval was given by the Uganda Virus Research Institute (reference number GC127), Uganda National Council for Science and Technology (reference number HS 1183), and London School of Hygiene and Tropical Medicine (reference number 6187). Written informed consent was received from all adults and emancipated minors and from parents or guardians for children; additional assent was obtained from children aged ≥8 years.

### Statistical Methods

For the parasitological survey, we anticipated that sampling 70 households per village would give >80% power to detect a 25% relative reduction in *S. mansoni* prevalence in the intensive trial arm compared with standard, assuming a coefficient of variation of 0.2 and standard arm *S. mansoni* prevalence of 35%. We also anticipated that this would give >80% power to detect an absolute increase of 10% in *S. mansoni* prevalence in the 13 intensively treated study villages between years 3 and 4, assuming a coefficient of variation of 0.1 for this analysis. For the test-of-cure substudy, we anticipated that screening 220 individuals in each arm would yield 70 Kato-Katz-positive participants in the standard arm and 55 in the intensive arm. Following up these individuals after treatment would give ~80% power to detect a difference in cure rate of 70% in the standard arm [21] vs 40% in the intensive arm.

Characteristics of parasitological survey participants and test-of-cure substudy participants were summarized. The effect of 4 years’ intensive vs standard anthelminthic intervention on *S. mansoni* prevalence was assessed using a cluster-level analysis, as described previously [15]. Briefly, the risk ratio was calculated as the mean of the cluster-specific proportions with *S. mansoni* infection in the intensive arm divided by the corresponding mean in the standard arm, with the 95% confidence interval (CI) calculated using a Taylor series approximation for the standard error and *P* values from unpaired *t* tests. The prevalence of *S. mansoni* infection was compared between time points using paired *t* tests of cluster-level proportions.

Cure rate was defined as the proportion of participants who were Kato-Katz negative on all 3 samples at follow-up, out of those positive for *S. mansoni* on at least 1 stool sample at baseline. Cure rates were compared between the standard and intensive arms using chi-square tests, with further comparisons adjusting for age, gender, and baseline infection intensity using logistic regression. Egg reduction rates were calculated as 100* (1 – [mean arithmetic egg count at follow-up/mean arithmetic egg count at baseline]) [22, 23], with 95% confidence intervals for ERRs and the difference between ERRs (intensive vs standard trial arms) calculated using a bias-corrected bootstrapping approach with 1000 permutations. Changes in CAA concentration between baseline and 4 weeks post-treatment were compared between the arms using mixed-effects linear regression, with an interaction term between time and trial arm fitted to assess whether the change over time differed between the 2 trial arms.

## RESULTS

### Parasitological Survey

Between March 21 and November 16, 2017, a total of 1790 households were randomly selected to take part in the parasitological survey (2 villages contained fewer than the target sample size of 70 households; in these villages, all households were included). Among these, a total of 2086 individuals, representing 1078 households, provided stool samples. The predominant reasons for not taking part were that the household was unoccupied or the occupants were absent upon repeated visits. The median (interquartile range) age of participants providing stool samples was 26 (8–36) years in the standard intervention arm and 27 (9–36) years in the intensive intervention arm; 52% and 51% were male in the standard and intensive arms, respectively.

**Figure 1.**
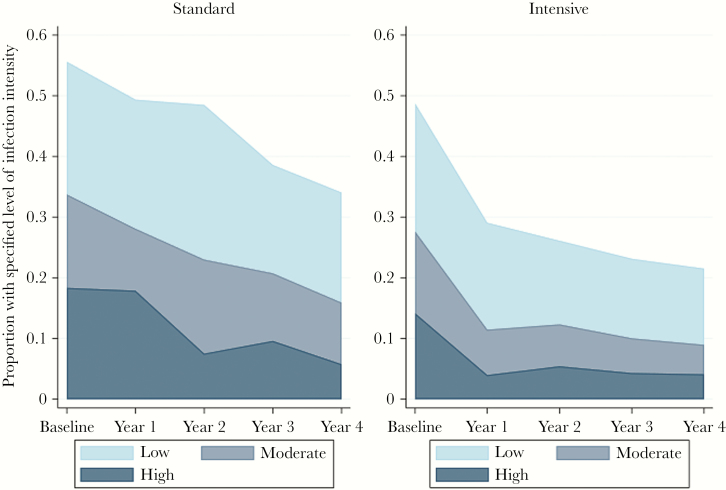
Prevalence and intensity of *Schistosoma mansoni* infection (as detected by Kato-Katz examination of a single stool sample) over 4 years of anthelminthic intervention, by trial arm.

The prevalence of *S. mansoni* based on Kato-Katz analysis of a single stool sample was 28% overall, 34% in the standard arm, and 22% in the intensive arm (risk ratio for effect of intensive vs standard anthelminthic intervention, 0.62; 95% confidence interval, 0.40–0.94; *P* = .02). Figure 1 shows the overall infection prevalence and the proportion with low, moderate, and heavy infections over the 4 years of intervention by treatment arm. The prevalence of *S. mansoni* infection in the intensive arm declined by a small amount during the fourth year of intervention (3-year prevalence, 23.1%; 4-year prevalence, 21.6%; *P* = .21), with a somewhat greater decline seen in the standard intervention arm (3-year prevalence, 38.6%; 4-year prevalence, 34.2%; *P* = .08). There was no statistical difference in these declines between trial arms (*P*_interaction_ = .47).

### Test-of-Cure Substudy

**Figure 2.**
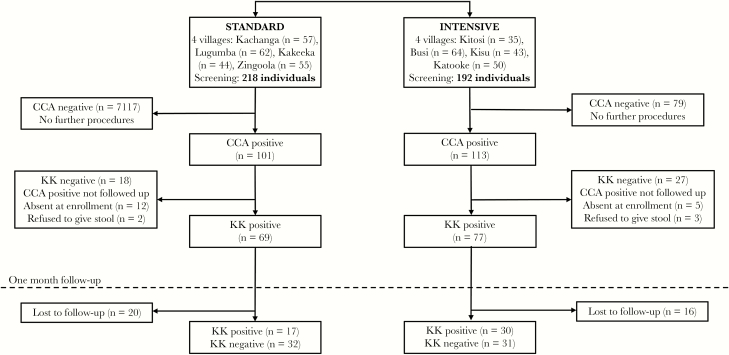
Flowchart of participants through cure rate study.

Figure 2 shows the flow of participants through the test-of-cure substudy. Of the 410 participants screened from 8 villages, 214 (52%) were positive by the urine CCA test, 101 (46%) of 218 screened in the standard trial arm and 113 (59%) of 192 screened in the intensive trial arm. Sixty-nine (68%) of the 101 CCA-positive participants in the standard arm were KK positive (on at least 1 of 3 stool samples) and enrolled for follow-up, of whom 49 (71%) were seen at follow-up and provided stool samples. Seventy-seven (68%) of the 113 CCA-positive participants in the intensive trial arm were KK positive (on at least 1 of 3 stool samples) and enrolled for follow-up, of whom 61 (79%) were seen and provided stool samples.

Characteristics of participants who were enrolled but not seen at follow-up and of those who were enrolled and followed up, stratified by trial arm, are shown in Table 1. Over two-thirds of enrolled participants were male, and the majority had contact with the lake every day. Successful follow-up in the cure rate substudy was higher for younger participants (preschool and school-aged) and somewhat lower for males than females. There were some differences between the standard and intensive trial arm participants for those who were followed up, with a higher proportion of females and school-aged participants in the intensive arm compared with the standard arm.

**Table 1.** Characteristics of Participants Enrolled and Followed up in the Cure Rate Study

				Seen at Follow-up and Included in Cure Rate Study Results		
Characteristic	Enrolled, not Followed up (n = 36), No. (%)	Enrolled, Followed up (n = 110), No. (%)	*P* Value^a^	Standard Arm (n = 49), No. (%)	Intensive Arm (n = 61), No. (%)	*P* Value^b^
Sex^c^						
Male (n = 100)	28 (28)	72 (72)		37 (77)	35 (57)	
Female (n = 45)	8 (18)	37 (82)	.187	11 (23)	26 (43)	.031
Age, y^c^						
<15 (n = 63)	8 (13)	55 (87)		19 (40)	36 (60)	
15–34 (n = 46)	15 (33)	31 (67)		18 (38)	13 (22)	
35 + (n = 35)	13 (37)	22 (63)	.010	11 (23)	11 (18)	.091
Main occupation^c^						
Child/student (n = 63)	7 (11)	56 (89)		19 (42)	37 (65)	
Fisherman (n = 41)	13 (32)	28 (68)		16 (36)	12 (21)	
Other (n = 34)	16 (47)	18 (53)	<.001	10 (22)	8 (14)	.073
Treated for malaria in past year^c^						
No (n = 69)	23 (33)	46 (67)		18 (44)	28 (54)	
Yes (n = 60)	13 (22)	47 (78)	.141	23 (56)	24 (46)	.341
Frequency of lake contact^c^						
Every day (n = 119)	29 (24)	90 (76)		40 (85)	50 (86)	
Less than every day (n = 22)	7 (32)	15 (68)	.462	7 (15)	8 (14)	.873
No. of praziquantel treatments in past 4 years^c^						
Mean (SD)	4.4 (3.2)	6.5 (4.3)	.010	4.0 (3.3)	7.9 (4.4)	<.001
Baseline infection intensity						
Light (n = 87)	22 (25)	65 (75)		26 (53)	39 (64)	
Moderate (n = 35)	8 (23)	27 (77)		14 (29)	13 (21)	
Heavy (n = 24)	6 (25)	18 (75)	.960	9 (18)	9 (15)	.511

^a^ *P* value for differences between those followed up and not followed up.

^b^ *P* value for differences between standard and intensive arm participants who were followed-up.

^c^Missing values for sex (1 participant), age (2 participants), occupation (8 participants), malaria treatment (17 participants), lake contact (5 participants), praziquantel treatment (18 participants).

**Figure 3.**
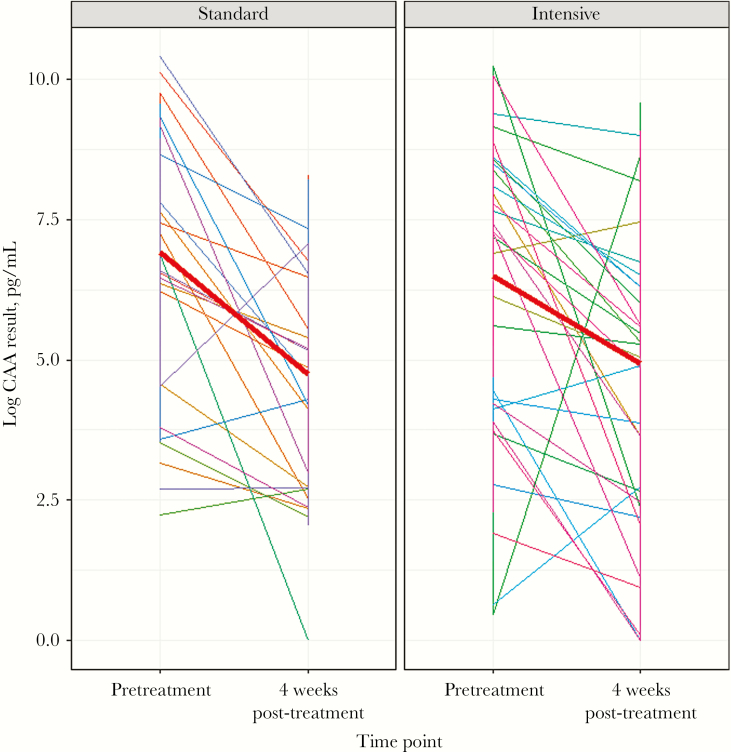
*Schistosoma mansoni* circulating anodic antigen (CAA) results at baseline and 4 weeks post-treatment among cure rate study participants, by trial arm. Each line represents a single participant; the thick red lines represent the median log CAA at each time point.

Table 2 shows CRs and ERRs stratified by trial arm. In the standard arm, 32 of the 49 participants seen at follow-up were KK negative on all 3 stool samples, yielding a cure rate of 65%. In the intensive arm, 31 of 61 seen at follow-up were KK negative, yielding a cure rate of 51% and a crude odds ratio (intensive vs standard arm) of 0.55 (95% CI, 0.25 to 1.19; *P* = .13). After adjusting for baseline infection intensity, sex, and age, the estimated odds ratio was 0.65 (95% CI, 0.27 to 1.58; *P* = .35). Egg reduction rates were 93.7% (95% CI, 84.9% to 97.7%) in the standard arm and 80.6% (95% CI, 43.8% to 93.7%) in the intensive arm, with the difference in ERR estimated as –13.1% (95% CI, –47.9% to 2.2%). CAA was measured in 90 participants (37 standard arm, 53 intensive arm) who provided sera at baseline and 4 weeks post-treatment. Figure 3 shows the log CAA concentration for cure rate study participants at baseline and 4 weeks post-treatment. In both trial arms, the median CAA concentration reduced following treatment, with a slightly smaller reduction seen in the intensive arm participants, but there was no statistical evidence of interaction (*P* = .35).

**Table 2.** Cure Rate and Egg Reduction Rate by Trial Arm

Outcome	Standard (Annual Praziquantel), No. (%) (95% CI)	Intensive (Quarterly Praziquantel), No. (%) (95% CI)	Unadjusted OR/Difference (95% CI)	Adjusted OR (95% CI)^a^
Cure rate	32/49 (65.3%) (50.4% to 78.3%)	31/61 (50.8%) (37.7% to 63.9%)	0.55 (0.25 to 1.19)	0.65 (0.27 to 1.58)
Egg reduction rate	93.7% (84.9% to 97.7%)	80.6% (43.8% to 93.7%)	–13.1% (–47.9% to 2.2%)	^b^

Abbreviations: CI, confidence interval; ERR, egg reduction rate; OR, odds ratio.

^a^Adjusted for baseline infection intensity, sex, and age.

^b^Adjusted results are not shown for ERR, as it is calculated at the population level.

## DISCUSSION

In these Lake Victoria island communities, *S. mansoni* prevalence in villages receiving 4 years of community-wide quarterly praziquantel treatment was 38% lower than in villages receiving annual treatment. The greatest reduction in *S. mansoni* prevalence in quarterly treated villages occurred in the first year of the intervention and was followed by smaller reductions in subsequent years and a plateau in prevalence of moderate–heavy infections, in contrast to annually treated villages where *S. mansoni* prevalence declined approximately linearly throughout the 4 years of intervention. Our formal test-of-cure indicated some suggestion of a reduction in cure rates and egg reduction rates among residents of quarterly treated villages compared with those in annually treated villages, although no difference was seen using the more sensitive CAA method.

Previous trials focusing on school children in a high-prevalence setting in Tanzania [24] and a low- to moderate-prevalence setting in Kenya [25] found no additional benefit of annual compared with biennial MDA for reducing prevalence. Our study suggests that more intensive (quarterly) treatment can reduce prevalence compared with an annual treatment strategy. However, after 4 years of intensive quarterly treatment with an average treatment coverage by treatment round of 63% [15], the prevalence of *S. mansoni* reduced from 49% at baseline to a still relatively high level of 22%, based on Kato-Katz analysis of a single stool sample. This is a relative reduction of 55%, demonstrating that our study setting might be considered a persistent hot spot [26].

In the cure rate substudy, the observed egg reduction rate of 80.6% was below the 90% threshold of optimal praziquantel efficacy set by the World Health Organization (although the 95% confidence interval does include 90%) [27], and there was some evidence that ERRs based on KK were reduced in villages that had received quarterly compared with annual praziquantel, although results from the more sensitive CAA test did not show evidence of such a reduction. Although convincing evidence for the broader emergence of resistance in countries where MDA has been used for many years is lacking, continued vigilance is needed to ensure that praziquantel remains an effective drug for the treatment of schistosomiasis.

A major limitation of the cure rate substudy was that the planned sample size was not achieved, resulting in reduced power to detect differences between the 2 trial arms. This was predominantly due to difficulties in locating cure rate study participants for follow-up investigations in this highly mobile study population, with particular difficulties in locating men of working age. Further studies based on larger sample sizes and parasite genetic analyses are required to further test this outcome. We were not able to assess re-infection rates in this study. However, as the follow-up period in this study was 1 month, it is unlikely that the ERRs and cure rates were influenced by re-infections given the life cycle of the parasite. We cannot rule out the possibility that immature parasites, not susceptible to praziquantel, may have been present at enrollment and subsequently matured, contributing to the infections detected at follow-up.

In conclusion, 4 years of quarterly praziquantel reduced schistosomiasis prevalence compared with annual treatment, although infection following both strategies remained common. Continued vigilance, for example, through periodic investigation of cure rate and ERR, should be maintained to ensure that parasites do not develop tolerance to praziquantel in ongoing MDA programs. Complementary or alternative strategies are needed to further reduce the schistosomiasis burden.
